# Progression of Contralateral Hearing Loss in Patients With Sporadic Vestibular Schwannoma

**DOI:** 10.3389/fneur.2020.00796

**Published:** 2020-08-14

**Authors:** Samuel Early, Charlotte E. Rinnooy Kan, Maura Eggink, Johan H. M. Frijns, Konstantina M. Stankovic

**Affiliations:** ^1^Eaton-Peabody Laboratories, Department of Otolaryngology—Head and Neck Surgery, Massachusetts Eye and Ear, Boston, MA, United States; ^2^Department of Otolaryngology—Head and Neck Surgery, Harvard Medical School, Boston, MA, United States; ^3^San Diego School of Medicine, University of California, San Diego, San Diego, CA, United States; ^4^Leiden University Medical Center, Leiden, Netherlands; ^5^University Medical Center Groningen, Groningen, Netherlands; ^6^Department of Otolaryngology—Head and Neck Surgery, Leiden University Medical Center, Leiden, Netherlands; ^7^Leiden Institute for Brain and Cognition, Leiden, Netherlands; ^8^Program in Speech and Hearing Bioscience and Technology, Harvard Medical School, Boston, MA, United States; ^9^Harvard Program in Therapeutic Science, Harvard Medical School, Boston, MA, United States

**Keywords:** vestibular schwannoma, hearing loss, contralateral, secreted factors, outcomes

## Abstract

**Background and Introduction:** Vestibular schwannomas (VSs) are the most common tumors of the cerebellopontine angle, typically presenting unilaterally with ipsilateral sensorineural hearing loss (SNHL). The mechanism of tumor-induced hearing loss has recently been shown to be related to secreted tumor factors, in addition to mechanical compression of the adjacent auditory nerve, and these factors may percolate through CSF or blood to affect contralateral hearing as well.

**Methods:** This is a retrospective study of medical records for patients treated for VS at Mass Eye and Ear from January 1994 through October 2018. Included patients had unilateral VS and sequential audiometry allowing for longitudinal assessment of hearing over time. Mass Eye and Ear's audiology database was used to select age- and sex-matched case controls, also with sequential audiometry, from the non-VS population. Subgroup analysis was performed by age, sex, baseline hearing, and tumor size at initial diagnosis. Hearing loss progression was performed using Kaplan-Meier analysis to account for variable follow-up times.

**Results:** A total of 661 patients were identified with VS and sequential audiometry. The population was predominantly female vs. male (368 vs. 293, *p* = 0.0035), driven primarily by younger patients with Koos 4 tumors (76 female vs. 49 male, *p* = 0.016). Patients with normal baseline hearing bilaterally (*N* = 241) demonstrated no significant difference in hearing loss progression in VS-contralateral vs. control ears. Patients with abnormal baseline VS-ipsilateral hearing (*N* = 190), however, demonstrated significantly higher likelihood of reaching moderate SNHL in VS-contralateral ears. Subgroup analysis by age, sex, and baseline tumor size did not yield any subgroup-specific trends for hearing loss progression.

**Discussion and Conclusion:** This is the largest study to date tracking long-term bilateral hearing outcomes in patients with VS, and demonstrates that, in patients with abnormal hearing in the VS-ipsilateral ear, there exists a long-term risk of progression to moderate hearing loss in the contralateral ear as well. Combined with the absence of significant changes in word understanding in the affected ears, these findings may provide clues to the nature of tumor-secreted factors involved in VS-associated hearing loss. Female predominance within the VS patient population is confirmed, driven mostly by younger female patients with Koos 4 tumors.

## Introduction

Vestibular schwannomas (VSs) are Schwann cell tumors that typically originate from the nerve sheath of a vestibular branch of the eighth cranial nerve within the internal auditory canal (IAC), and in 95% of patients cause sensorineural hearing loss (SNHL) (1, 2). As these tumors grow, they extend into the cerebellopontine angle (CPA) where they account for 80% of tumors in that location (3). The incidence of VSs ranges from one out of 10,000 for unilateral and sporadic VS to 1 per 30,000 for bilateral VSs, occurring in association with neurofibromatosis type 2 syndrome (4, 5). In addition to SNHL, VSs can cause tinnitus, balance problems, and cranial neuropathies such as facial paralysis, and as such have the potential to severely affect a patient's quality of life despite their nominally non-malignant nature. In large tumors that are proximal to the brainstem, VS tumors can be associated with life threatening complications of brainstem compression and hydrocephalus (3, 6). To date, no drug has been FDA approved to treat VS, limiting management options for VS to watchful waiting, surgery and radiotherapy (7). Both surgery and radiotherapy are associated with significant risks, including loss of hearing and facial nerve paralysis, with as many as 28% of patients experiencing at least one of these complications (8).

Despite VS being an important cause of human SNHL, the underlying pathophysiological mechanisms causing SNHL in patients with VS tumors are still incompletely understood (6). Early hypotheses that VSs cause SNHL by gradually increasing mechanical compression to the adjacent auditory nerve do not explain the lack of correlation between either radiographic tumor size or tumor extension into the IAC with changes in conventional audiometric criteria (9, 10). Additionally, audiometric threshold shifts are observed in patients without radiographic tumor growth (9). These clinical observations suggest additional explanations for the pathophysiology of SNHL, beyond only mechanical compression of adjacent structures. Recent studies have revealed new insights about the capacity of secreted factors, such as tumor necrosis factor alpha (TNFα), and extracellular vesicles (EVs) from human VSs to cause cochlear damage (11–16). The greater the severity of SNHL associated with a VS tumor, the greater the degree of hair cell loss and neuronal fiber disorganization seen in cochlear explants exposed to tumor secretions (11, 13). In addition, the degree of SNHL tends to be greater in patients whose tumors overexpress the NLRP3 inflammasome and the associated ototoxic molecules such as IL1β (12). These findings suggest secreted mechanisms for SNHL in patients with VS, independent of mechanical compression of the cochlear nerve.

Secretions produced by sporadic VS tumors could have the potential to reach the contralateral ear by percolating through cerebrospinal fluid (CSF) or blood. Because the vast majority of VS patients suffer from SNHL in the ipsilateral ear, any potential effect of tumor secretions on contralateral hearing is of high clinical relevance. Until now, no study has been done with a large enough patient population, incorporating sufficiently comprehensive audiometric data, to investigate the possible effect of sporadic VS on hearing in the contralateral ear. This study examines whether in patients with unilateral sporadic VS, the progression of SNHL in the contralateral ear is faster than would be otherwise expected for unrelated age-related hearing loss in the general population.

## Materials and Methods

A retrospective chart review was performed of patients diagnosed with VS from January 1994 to October 2018 at Massachusetts Eye and Ear. Institutional Review Board (IRB) approval was obtained from the Human Studies Committee at Massachusetts Eye and Ear and Massachusetts General Hospital (IRB 16-103H).

### Patient Selection

**Figure 1:**
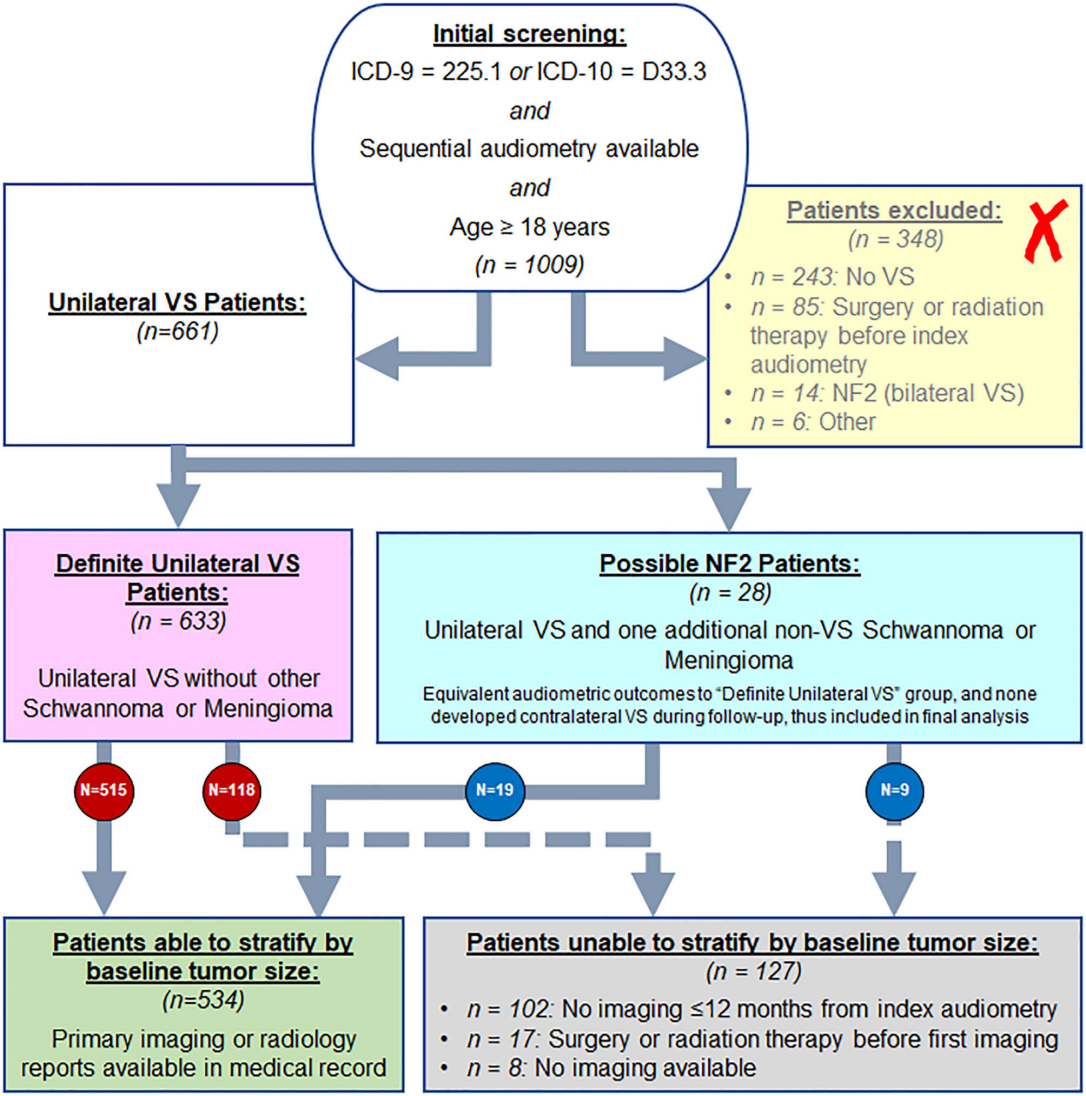
Screening approach from Mass Eye and Ear audiology database for VS patients to include in study, as described in methods.

Review of electronic medical and billing records at Mass Eye and Ear identified 1009 patients aged 18 years and older with diagnosis codes consistent with VS: 225.1 (ICD-9) and D33.3 (ICD-10), and who additionally had undergone sequential audiometry at Mass Eye and Ear, in order to permit analysis of longitudinal changes in hearing. Patient charts and imaging were reviewed to confirm absence of bilateral VS or three or more total schwannomas or meningiomas, to avoid inclusion of any patients with NF2 (Figure 1). Patients were additionally excluded if they had undergone surgery or radiation therapy before first available audiometry, or if they had pre-existing profound VS-contralateral hearing loss or deafness at time of earliest audiometry.

After defining the VS study population, an age- and sex-matched case control population was selected at a 3:1 ratio from the general patient population at Mass Eye and Ear; patients in the case control population were required to have a diagnosis code of SNHL or mixed hearing loss and sequential audiometry performed at Mass Eye and Ear, without any other known otologic disease and with normal hearing at baseline in at least one ear. When >3 case control candidates were available, priority for inclusion was given to patients for whom frequency of audiometric testing most closely matched their assigned VS patient. All case control patients with significant change in hearing for either ear at any time during follow-up (increase in thresholds to >40 dB from the normal range at baseline, or decline in word understanding to <78% from the normal range at baseline) were further reviewed in detail for any history of occult otologic disease, and excluded from the case control group if identified by chart review. Each case control patient had normal hearing in at least one ear, with either one or both ears included for analysis depending on baseline hearing status.

Kaplan-Meier analysis was performed to evaluate survival with endpoint defined by set audiometric criteria; ipsilateral ears were censored if undergoing either surgery or radiation therapy. Log rank test was applied to evaluate significance in rate of endpoint achievement between VS-ipsilateral ears and VS-contralateral ears, and between VS-contralateral ears and case control group ears. All analyses were performed in Matlab (Mathworks, Natick, MA) and Excel (Microsoft Corporation, Redmond, WA).

### Audiometry

VS patients were stratified by baseline audiometry; audiologic measurements included speech audiometry [word recognition score (WRS%)] and threshold audiometry (dB HL). Word recognition score was calculated using standardized word list of monosyllables, measured as a percentage of correctly recognized words after listening to a recorded word list in quiet, typically at 70 dB or at the level at which the patient's speech intelligibility curve plateaus. Pure tone average (PTA) threshold was calculated as a three-tone average of bone conduction thresholds across 500 Hz, 1 kHz, and 2 kHz frequencies as the primary threshold-based hearing metric; alternate methods such as including the 4 kHz frequency within a four-tone average, and use of AAO-HNS hearing classification were also employed secondarily to confirm findings and trends. Normal baseline hearing was defined as WRS ≥ 92% and a pure-tone average (PTA) threshold of ≤ 25 dB assessed by bone conduction; additionally, if a patient's initial audiometry was performed in the setting of sudden SNHL, however subsequent audiometry demonstrated return to normal hearing within 90 days, then baseline hearing was considered normal for purposes of baseline hearing assignment. If an ear had normal baseline air thresholds but was not assessed for bone thresholds, then baseline hearing was also assigned as normal.

Progression of audiometric changes were assessed both ipsilateral and contralateral to known VS tumors. Progression of hearing loss over time was evaluated using Kaplan-Meier analysis, with change in hearing used to define endpoint. For PTAs, the primary assigned endpoint was defined as moderate hearing loss (PTA > 40 dB), with secondary endpoint of moderately severe hearing loss (PTA > 55 dB) per definition by the American Speech-Language-Hearing Association. For WRS%, the primary endpoint was defined as WRS < 78% (significant decline from baseline normal hearing at ≥92%), with secondary endpoint defined as word understanding <60% (significant further decline from the primary endpoint, and also clinical threshold for consideration of cochlear implantation) (17, 18).

### Tumor Size

Review of tumor sizes at the time of each patient's index audiometry was performed via interpretation of primary imaging or review of imaging reports, depending on availability within the medical record. In cases where neither primary imaging nor reports were available, patients were included for analysis as part of the entire population, but not for analysis stratified by tumor size. Given that VS tumors typically grow slowly, at an average of 1–2 mm/year, and given that changes <1 mm are within the margin of error for historically available imaging resolution, imaging was considered representative of baseline tumor size if completed within 12 months of the patient's first available audiogram (19, 20).

### Koos Classification

Tumor extent was assessed according to the Koos grading system. Tumors were divided into various classes, described as Koos 1 (purely intrameatal), Koos 2 (intrameatal and extrameatal, but no contact with the brainstem), Koos 3 (touching the brainstem without compression of the brainstem), or Koos 4 (touching the brainstem with compression of the brainstem) (21). For patients where neither contemporary imaging reports nor primary images were available from within 12 months of initial audiometry, these patients were included in analysis of the general patient population but could not be included in Koos segmentation.

## Results

Figure 1 shows the results of analysis of 1,009 patients with billing codes consistent with unilateral VS. A total of 257 patients were found to have either no VS (*N* = 243) or were found to have NF2 (*N* = 14) and were therefore excluded. Patients with no VS most frequently had a meningioma (*N* = 39), facial nerve schwannoma (*N* = 29) or intrachochlear schwannoma (*N* = 18). Furthermore, 25 patients had SNHL, seven had labyrinthine schwannoma, five had Meniere's disease, two had benign paroxysmal positional vertigo, and one had vestibular neuritis; none of these had imaging or audiometric evidence of retrocochlear pathology. An additional 117 cases had no evidence of any pathology involving the labyrinthine system, facial nerve, or vestibulocochlear nerve. Eighty-five patients were excluded due to existence of prior surgery or radiation in the area of the cochlear nerve preceding first audiometry. Six patients were excluded due to other reasons: three patients with glioma in addition to VS, one patient with Von Hippel-Lindau syndrome in addition to VS, and two patients with known long-standing contralateral deafness at time of index audiometry (thus precluding evaluation of changes in contralateral hearing). Patients who did not meet criteria for NF2 but had one additional non-VS schwannoma or meningioma in addition to a unilateral VS (*N* = 28) were analyzed separately from the included patient population in order to compare these patients with suspected NF2, but not meeting strict criteria, with the general VS patient population, with plan to exclude them from final analysis if significant differences in outcomes were found (22).

After the exclusions noted above, a core population of 661 patients diagnosed with unilateral VS were identified and described in Table 1. Median age was 56 years ranging from 21 to 89 years (standard deviation 12.6 years). Regarding tumor laterality, 320 (48.4%) tumors were right sided and 341 (51.6%) tumors were left sided. A significant female predominance was seen with 368 (55.7%) females compared to 293 (44.3%) males (*p* = 0.0035). Of all patients with unilateral VS, 274 patients (41.5%) eventually received surgery alone as treatment, 104 patients (15.7%) received radiotherapy alone, 14 patients (2.1%) received both surgery and radiotherapy and in 269 patients (40.7%) the tumor was monitored through watchful waiting without any surgical or radiologic intervention. In total, 534 patients had imaging within 12 months of index audiometry, and 518 within 6 months. The median tumor size was 12 mm, with a minimum of 1.5 mm to a maximum of 52 mm (standard deviation 7.9 mm). The remaining 127 patients could not be stratified by tumor size—these patients either had no imaging records available within 12 months of index audiometry or had undergone surgical or radiation treatment before the earliest available imaging. Patients with one additional non-VS schwannoma or meningioma were significantly older (60.8 vs. 55.2 years, *p* = 0.021) and had smaller average tumor size (9.0 vs. 13.1 mm, *p* = 0.026), however did not differ compared to the general population in terms of sex distribution, tumor laterality, or treatment modality.

**Table 1:** Demographics in patients with sporadic unilateral VS.

	**Unilateral VS patients**							***p*-value for interaction, “Yes vs. No:”**
	**With other non-VS schwannoma or meningioma?**	**Overall**		**No**		**Yes**		
Patient number	*N*	661		633		28		
Age (years)	Mean	55.4		55.2		60.8		*P* = 0.021
	Median	56		55		62		
	Range	21–89		21–89		37–78		
	Std. Dev.	12.6		12.7		10.6		
Tumor laterality	Right:	320	*p* = 0.41	307		13		*p* = 0.83
	Left:	341		326		15		
Sex	Male	293	*p* = 0.0035	283		10		*p* = 0.48
	Female	368		350		18		
Tumor dimensions (n = 531 patients with available baseline imaging ≤ 12 months from index audiometry that was sufficient to determine max tumor dimension^*^)	Max Dimension (mm)	*(n = 531)*		*(n = 512)*		*(n = 19)*		*p* = 0.026
	- Mean	13.0		13.1		9.0		
	- Median	12		12		7.5		
	- Range	1.5–52		1.5–52		3–24		
	- Std. Dev.	7.9		7.9		5.6		
	Tumor size range:							
	≤10 mm	215	(40.5%)	202	(39.5%)	13	(68.4%)	*p* = 0.013
	>10–20 mm	235	(44.2%)	230	(44.9%)	5	(26.3%)	
	>20 mm	81	(15.3%)	80	(15.6%)	1	(5.3%)	
Tumor treatment modality	Surgery only	274	(41.5%)	266	(42.0%)	8	(28.6%)	*p* = 0.40
	Radiation only	104	(15.7%)	100	(15.8%)	4	(14.3%)	
	Both surgery and radiation	14	(2.1%)	13	(2.1%)	1	(3.6%)	
	No therapy	269	(40.7%)	254	(40.1%)	15	(53.5%)	

* *For three patients with available baseline imaging and without other non-VS schwannoma or meningioma, chart review was sufficient only to determine Koos classification but not maximum tumor dimension*.

Of the 534 patients with contemporary baseline imaging available, 511 could be stratified by their tumor's Koos classification as shown in Table 2. The age of Koos 4 patients was found to be significantly lower (*p* = 0.0012) than in smaller tumors. Significance for female vs. male predominance in the population was only seen in Koos 4 tumors as well (76 female vs. 49 male, *p* = 0.016). Increasing Koos classification was associated with larger tumors and increasing likelihood of surgical intervention, while patients with lower Koos classification had smaller tumors and were more likely to be managed without either surgery or radiotherapy. Radiation therapy was most commonly applied to patients with Koos 2 and Koos 3 tumors; multimodal therapy, the least common approach, was primarily reserved for Koos 4 tumors.

**Table 2:** Tumor dimensions in patients with sporadic unilateral VS.

	**Unilateral VS Patients with available baseline imaging sufficient for assessment by Koos classification (n = 511)**								
	**Koos classification:**	**Koos 1**		**Koos 2**		**Koos 3**		**Koos 4**	
Patient number	*N*	214		149		23		125	
Age (years)^*^	Mean	56.5		56.6		56.5		52.4	
	Median	57.5		57		56		53	
	Range	21–87		24–81		40–74		22–79	
	Std. Dev.	12.8		12.2		11.0		12.7	
Tumor laterality	Right:	101	*p* = 0.41	79	*p* = 0.46	11	*p* = 0.83	59	*p* = 0.53
	Left:	113		70		12		66	
Sex	Male	93	*p* = 0.056	72	*p* = 0.68	10	*p* = 0.53	49	*p* = 0.016
	Female	121		77		13		76	
Tumor dimensions^**^	Max Dimension (mm)	*(n = 213)*		*(n = 149)*		*(n = 22)*		*(n = 124)*	
	- Mean	6.4		13.2		16.1		23.3	
	- Median	6		13		15		22	
	- Range	1.5–14		4–28		11–25		12–52	
	- Std. Dev.	3.0		3.2		4.0		7.1	
	Tumor size range:								
	≤10 mm	189	(88.7%)	22	(14.8%)	0	(0.0%)	0	(0.0%)
	>10–20 mm	24	(11.3%)	124	(83.2%)	18	(81.8%)	52	(41.9%)
	>20 mm	0	(0.0%)	3	(2.0%)	4	(18.2%)	72	(58.1%)
Tumor treatment modality	Surgery only	37	(17.3%)	66	(44.3%)	14	(60.9%)	95	(76.0%)
	Radiation only	31	(14.5%)	33	(22.1%)	6	(26.1%)	17	(13.6%)
	Both surgery and radiation	3	(1.4%)	1	(0.7%)	0	(0.0%)	5	(4.0%)
	No therapy	143	(66.8%)	49	(32.9%)	3	(13.0%)	8	(6.4%)

* *Significant (p = 0.0012) difference in age when comparing Koos 4 vs. all other tumors; non-significant difference in age distribution between Koos 1, 2, and 3 classifications*.

** *For one each of patients with Koos 1, Koos 3, and Koos 4 tumors, chart review was sufficient to determine Koos classification but not maximum tumor dimension*.

**Figure 2:**
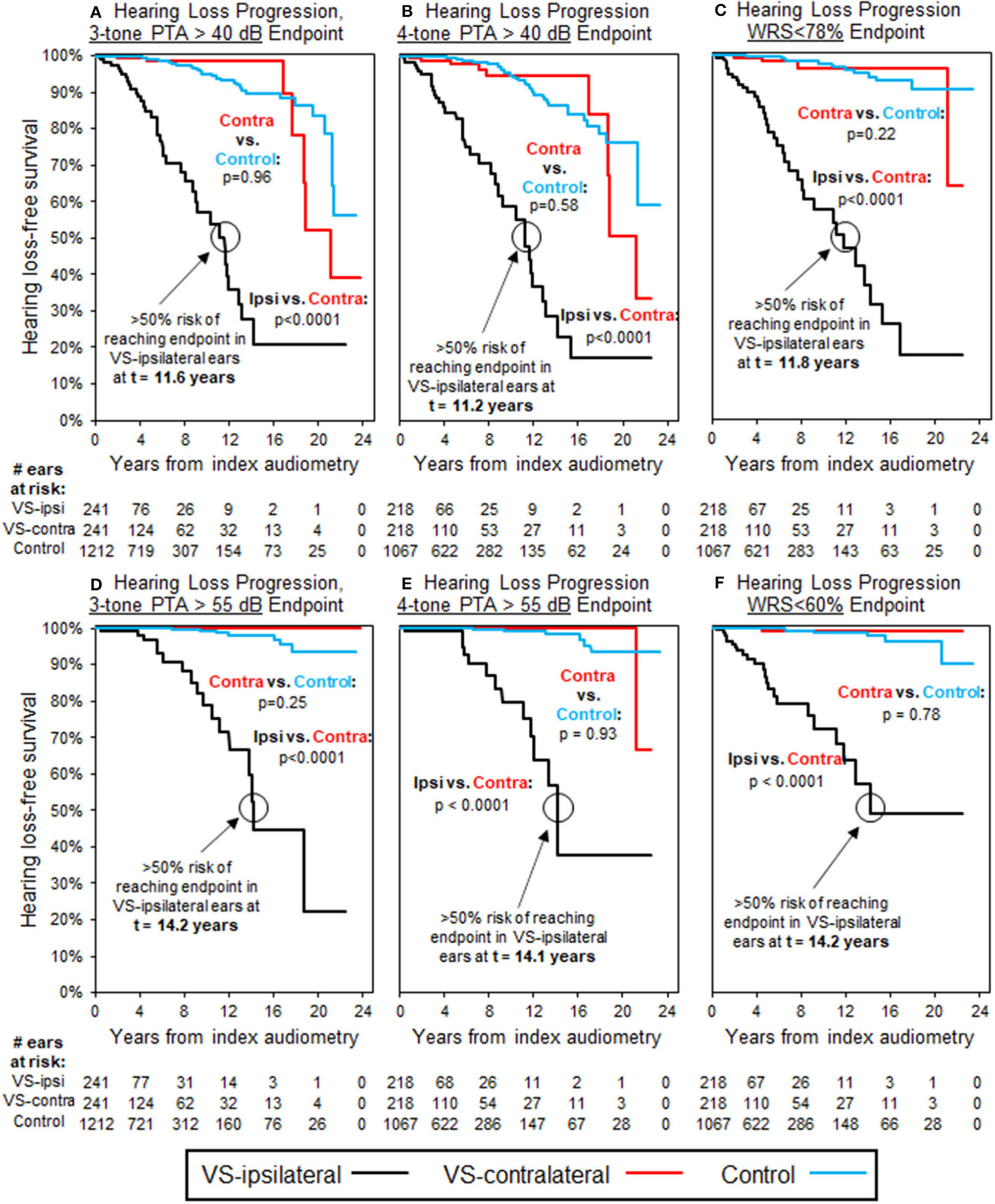
Kaplan-Meier curves for HL progression in patients with baseline normal hearing bilaterally (WRS ≥ 92% and PTA ≤ 25 dB bilaterally); see Table 3 for demographics. Analysis uses three-tone PTA to assess baseline hearing in **(A,D)**, as well as endpoint in **(A)**, while four-tone PTA used, respectively in **(B,C,E,F)**. Endpoint bone three-tone PTA > 40 dB (moderate HL); **(B)** Endpoint bone four-tone PTA > 40 dB (moderate HL); **(C)** Endpoint word understanding <78%; **(D)** Endpoint bone three-tone PTA > 55 dB (moderately severe HL); **(E)** Endpoint bone four-tone PTA > 55 dB (moderately severe HL); **(F)** Endpoint word understanding <60%. All control ears with normal baseline hearing defined by same criteria as for VS-ipsilateral and contralateral ears.

Baseline audiometric analysis, as shown in Table 3, demonstrated 310 patients with normal three-tone PTAs (≤25 dB) bilaterally at baseline, 257 of which additionally were normal by four-tone PTA assessment; 298 patients had normal word understanding (WRS ≥ 92%) bilaterally. Furthermore, a total of 241 patients demonstrated both normal three-tone PTAs and word understanding bilaterally at baseline, while 218 demonstrated both normal four-tone PTAs and word understanding bilaterally. Average interval of follow-up audiometry was 2.1 years, and the selection of age- and sex-matched case controls, as described in Methods, resulted in average interval of follow-up audiometry also of 2.1 years. Results of Kaplan-Meier survival analysis in these patients demonstrated significant decline in hearing, both in terms of thresholds and word understanding, in VS-ipsilateral ears over time, regardless of endpoint chosen (Figure 2). The median time to reach threshold endpoint in VS-ipsilateral ears was 11.6 years for moderate SNHL by three-tone PTA criteria, and 11.2 years by four-tone PTA criteria, while for moderately severe SNHL the median time to reach endpoint was 14.2 years by three-tone PTA criteria and 14.1 years by four-tone PTA criteria; median time to reach word understanding endpoint in VS-ipsilateral ears was 11.8 years for WRS < 78% and 14.2 years for WRS < 60%. No significant difference was observed in hearing loss progression between VS-contralateral ears and age- and sex-matched controls in these patients, regardless of endpoint (moderate SNHL, moderately severe SNHL, WRS < 78%, or WRS < 60%).

**Table 3:** Demographics in patients with sporadic unilateral VS, segmented by baseline VS-ipsilateral hearing.

	**Normal baseline hearing in VS-ipsilateral ear**					**Abnormal baseline hearing in VS-ipsilateral ear**				
	**PTA ≤ 25 dB**		**WRS ≥ 92%**	**PTA ≤ 25 dB and WRS ≥ 92%**		**PTA > 25 dB**		**WRS < 92%**	**PTA > 25 dB and WRS < 92%**	
	**3-tone**	**4-tone**		**3-tone PTA**	**4-tone PTA**	**3-tone**	**4-tone**		**3-tone PTA**	**4-tone PTA**
Total *N*	310	257	298	241	218	269	290	270	190	204
Mean Age (years)^*^	51.0	50.0	51.6	50.4	49.8	57.1	56.2	56.7	57.1	56.0
Male *N*	131	100	124	101	87	118	129	122	83	90
(%)	(42.3%)	(38.9%)	(41.6%)	(41.9%)	(39.9%)	(43.9%)	(44.5%)	(45.2%)	(43.7%)	(44.1%)
Female *N*	179	157	174	140	131	151	161	148	107	114
(%)	(57.7%)	(61.1%)	(58.4%)	(58.1%)	(60.1%)	(56.1%)	(55.5%)	(54.8%)	(56.3%)	(55.9%)

**Figure 3:**
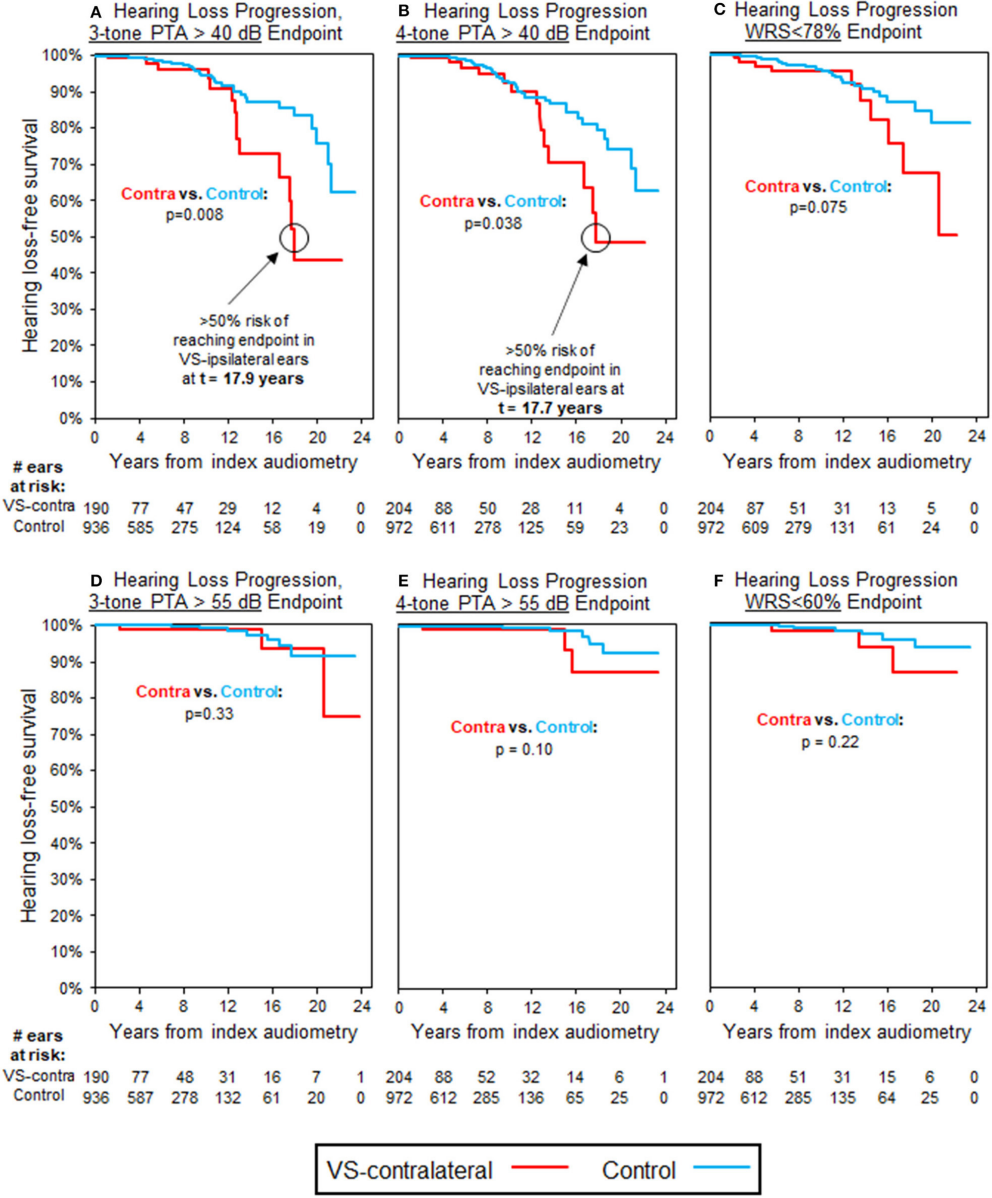
Kaplan-Meier curves for HL progression in patients with baseline abnormal hearing in VS-ipsilateral ear (WRS < 92% and PTA > 25 dB) and baseline normal hearing in VS-contralateral ear (WRS ≥ 92% and PTA ≤ 25 dB); see Table 3 for demographics. Analysis uses three-tone PTA to assess baseline hearing in **(A,D)**, as well as endpoint in **(A)**, while four-tone PTA used, respectively in **(B,C,E,F)**. **(A)** Endpoint bone three-tone PTA > 40 dB (moderate HL); **(B)** Endpoint bone four-tone PTA > 40 dB (moderate HL); **(C)** Endpoint word understanding <78%; **(D)** Endpoint bone three-tone PTA > 55 dB (moderately severe HL); **(E)** Endpoint bone four-tone PTA > 55 dB (moderately severe HL); **(F)** Endpoint word understanding <60%. All control ears with normal baseline hearing defined by same criteria as for VS-ipsilateral and contralateral ears.

* *Significant (p < 0.0001) difference in age between patients with VS-ipsilateral normal vs. abnormal hearing at baseline, regardless of whether measured by PTAs, WRS, or both. Outputs plotted in Figures 2, 3 are based on the most restrictive hearing definitions for normal vs. abnormal baseline hearing, as outlined above by red boxes. No significant differences were found in sex distribution, tumor laterality or tumor size for any group compared to general study population. All patients with normal VS-contralateral hearing at baseline*.

Table 3 also demonstrates that 269 patients with normal three-tone PTAs (≤ 25 dB) in the VS-contralateral ear had abnormal three-tone PTAs (>25 dB) in the VS-ipsilateral ear at baseline, while 290 patients with normal four-tone PTAs (≤25 dB) in the VS-contralateral ear had abnormal four-tone PTAs (>25 dB) in the VS-ipsilateral ear at baseline; 270 patients with normal word understanding (WRS ≥ 92%) in the VS-contralateral ear had abnormal word understanding (WRS < 92%) in the VS-ipsilateral ear at baseline. In total, 190 patients had both normal three-tone PTAs and normal word understanding in the VS-contralateral ear, and also had both abnormal three-tone PTAs and abnormal word understanding in the VS-ipsilateral ear at baseline; 204 patients had both normal four-tone PTAs and normal word understanding in the VS-contralateral ear, and also had both abnormal four-tone PTAs and abnormal word understanding in the VS-ipsilateral ear at baseline. Average interval of follow-up audiometry was 2.1 years; selection of age- and sex-matched case controls, as described in Methods, resulted in average interval of follow-up audiometry also of 2.1 years. Results of Kaplan-Meier survival analysis in these patients demonstrated significant decline in VS-contralateral hearing using moderate SNHL as endpoint, either by three-tone PTA criteria (*p* = 0.008) or four-tone PTA criteria (*p* = 0.038). Median time to endpoint was 17.9 years in VS-contralateral ears by three-tone PTA criteria, and 17.7 years by four-tone PTA criteria (Figures 3A,B). No significant difference was observed in hearing loss progression between VS-contralateral ears and age- and sex-matched controls when using other endpoints (moderately severe HL by either three- or four-tone PTA criteria, WRS < 78% or WRS < 60%), however the trend was consistently in the same direction regardless of endpoint used (Figures 3C–F). In these patients with abnormal baseline hearing in the VS-ipsilateral ear, progression of hearing loss in VS-ipsilateral ears could not be assessed.

Further subgroup analysis by patient age, sex, and Koos classification did not demonstrate any significant differences from the population-wide trends noted previously. Sub-segmentation of patients with abnormal hearing in the VS-ipsilateral ear by degree of hearing loss, whether by thresholds or word understanding, did not demonstrate any trend toward increased risk in the VS-contralateral ear with worsening VS-ipsilateral hearing status. Although a small group, the 28 patients with an additional non-VS schwannoma or meningioma did not show any significant differences in hearing loss progression, either by thresholds or word understanding, compared to the general patient population, and as such were included in the overall population analysis. Patient age, sex, and tumor size were not associated with any patterns of hearing loss progression in VS-contralateral ears, whether by thresholds or by change in word understanding.

Table 4 evaluates the significance of sex distribution based on a combination of both baseline hearing status and Koos classification in patients with baseline normal VS-contralateral hearing. Patients with Koos 4 tumors and baseline normal VS-ipsilateral hearing were exceptionally more likely to be female (72.2 vs. 27.8%, *p* = 0.0077) than other patient demographics, while distribution of the sexes did not differ when considering patients with Koos 4 classification and baseline abnormal VS-ipsilateral hearing, or in patients with Koos 1–3 tumor classification regardless of baseline hearing status.

**Table 4:** Sex distribution in patients with sporadic unilateral VS, segmented by baseline hearing and tumor Koos classification.

	**Patients with normal VS-ipsilateral baseline hearing** (**3-tone PTA ≤ 25 dB and WRS ≥ 92%**)				**Patients with abnormal VS-ipsilateral baseline hearing** (**3-tone PTA > 25 dB and WRS < 92%**)			
	**Koos 1**	**Koos 2**	**Koos 3**	**Koos 4**	**Koos 1**	**Koos 2**	**Koos 3**	**Koos 4**
Total *N*	89	43	8	36	40	59	7	36
Male *N*	41	21	3	10	16	25	3	15
(%)	(46.1%)	(48.8%)	(37.5%)	(27.8%)	(40.0%)	(42.4%)	(42.9%)	(41.7%)
Female *N*	48	22	5	26	24	34	4	21
(%)	(53.9%)	(51.2%)	(62.5%)	(72.2%)	(60.0%)	(57.6%)	(57.1%)	(58.3%)
*P*-value (M vs. F)	*p* = 0.46	*p* = 0.88	*p* = 0.48	*p* = 0.0077	*p* = 0.21	*p* = 0.24	*p* = 0.71	*p* = 0.32

*All patients with normal VS-contralateral hearing at baseline*.

Evaluation of all of the above hearing outcomes by alternate PTA metrics, for example AAO-HNS hearing classification groups, revealed the same trends toward hearing loss over time as the analysis performed using either three- or four-tone PTAs.

## Discussion

### Findings

This retrospective study demonstrates that in patients with unilateral VS and baseline abnormal hearing in the VS-ipsilateral ear, the progression of SNHL in the contralateral ear is significantly greater than expected for unrelated age-associated hearing loss in the general population. This finding is limited only to progression of thresholds to the level of moderate hearing loss, however directionally similar non-significant trends are also noted for other threshold- and word understanding-based endpoints. This interesting finding needs further examination in a future prospective study using a large number of patients with carefully documented otologic histories and sound exposures, as the finding suggests that VS-secreted factors may affect hearing in the contralateral ear. In the meantime, an alternative explanation for our finding could also be that patients with baseline abnormal VS-ipsilateral hearing and accelerated hearing loss in the VS-contralateral ear have an underlying process that expedites bilateral hearing loss, such as history of unreported prior audiotrauma. Findings regarding progression of VS-ipsilateral hearing loss in patients with baseline normal hearing are in line with previous research (23). The implications of these findings are significant both clinically, for the setting of expectations for long-term hearing prognosis in patients newly diagnosed with VS, and for basic science research to better understand the underlying pathophysiology of these tumors.

For the majority of VS patients in our study, progression of VS-contralateral hearing loss is significantly delayed and of a less-severe degree than in the VS-ipsilateral ear. This suggests that if percolating VS-secreted factors or extracellular vesicles do reach the contralateral ear, they must either suffer some level of degradation during passage through CSF or blood, or become too diluted to exert the full-strength ototoxic effect beyond a certain distance from the tumor. It is also possible that an immune mediated mechanism could play a role in the pathophysiology of hearing loss progression, as immune and inflammatory mechanisms have previously been found to occur in other inner ear pathologies such as Ménière's disease, DFNA34 hearing loss and related autoimmune and autoinflammatory diseases of the inner ear (24–26). A growing field of evidence suggests that inflammation is a key feature of the VS microenvironment as well, such as excessive activation of the NLRP3 inflammasome leading to upregulation of associated proteins NLRP3 and IL-1β, which have been preferentially upregulated in tumors associated with increased hearing loss (12, 27). The relative impact on thresholds vs. word understanding in contralateral ears can provide clues to the nature of which VS-secreted factors or immune mediated responses may be responsible for causing hearing loss in the VS-ipsilateral ear, and can guide further research in this direction.

Either an inflammatory or immune-mediated pathway would have the potential to stimulate innate immune responses against the contralateral ear, however it is uncertain whether this effect would be consistent with changes in contralateral hearing observed. The classic finding for hearing loss caused by VS tumors would be loss of word understanding out of proportion to change in thresholds, which we observe in ipsilateral ears. However, in the contralateral ear we find that threshold change rather than change in word understanding is most significant. The shape of KM curves in Figure 3, for threshold and word understanding endpoints, respectively, are very similar, but separation of trend curves does not become noticeable until >12 years of follow-up in either group—by this time, only 32/204 (15.7%) VS patients have reliable follow-up, with the remainder having either already reached endpoint or been censored. This smaller population sample size limits the ability to detect significance, but more significant trends in word understanding might still be found with longer follow-up or a larger study population.

Of key clinical significance, the lack of effect regarding higher-threshold endpoints or word understanding on contralateral hearing is a positive clinical indicator in patients with VS, and can provide some additional reassurance to patients in whom the contralateral ear is often their only hearing ear. In patients with normal baseline hearing in the VS-ipsilateral ear, assurance can be provided that no effect is seen on VS-contralateral hearing by any metric, however it would still be important to update patient expectations with any change in clinical status over time. Five patients with normal baseline hearing bilaterally did reach moderate VS-contralateral hearing loss at ≥17 years' follow-up (Figure 2A), however in all cases the VS-ipsilateral ear in these patients had either previously transitioned to abnormal hearing, had undergone radiation therapy, or had undergone surgical intervention with incomplete resection. While baseline normal VS-ipsilateral hearing does seem to be a protective factor against long-term hearing loss contralaterally, this effect does not appear to persist if hearing status in the VS-ipsilateral ear declines significantly.

Our study reveals a significant female predominance of VS, which is in line with female predominance for VS that has also been seen in previous retrospective studies (28–30). A possible explanation for the recurrent finding could be sex hormonal influence on the development of VS (31–33). We note as well that the difference in sex distribution is driven primarily by greater female prevalence in patients with Koos 4 tumors, that patients with Koos 4 tumors and baseline normal hearing have significantly younger average age in general, and that patients with normal baseline hearing in the VS-ipsilateral ear are also slightly more likely to be female. Particularly in patients with Koos 4 tumors and normal baseline hearing bilaterally, 72.2% of patients are female, compared to 55.7% in the study population as a whole. It may be that Koos 4 tumors, being physically larger and thus more likely to cause symptoms due to mechanical compression, are more likely to be diagnosed early relative to their effect on hearing through secreted factors. This presentation does not, however, explain the greater female predominance among large tumors with preserved hearing; rather, it may indicate a unique underlying pathophysiology for these large, brainstem-compressing tumors in younger female patients. A possible molecule mediating this effect and worthy of future investigation is fibroblast growth factor 2 (FGF2), because FGF2 is a mitogen, its serum levels are significantly higher in women than men without VS, and sporadic VS that secrete high levels of FGF2 have previously been associated with better hearing than VS that secrete low levels of FGF2 irrespective of tumor size (34, 35).

Within the four Koos classification groups the age of Koos 4 patients was found to be significantly lower than in other groups (*p* = 0.0012). When evaluating tumor size in other patient groups, however, no correlation is observed between tumor size and patient age, and no correlation between tumor size and progression of hearing loss either ipsi- or contralaterally. Evaluation of long-term hearing in patients with Koos 4 tumors is difficult since most patients undergo either surgery or radiation therapy very soon after diagnosis, after which time they are less likely to continue with long-term audiometric follow-up. Patients with smaller tumors that do not compress the brainstem are more likely to be found incidentally or with mild symptoms, and are more likely to be followed without immediate intervention. Within Koos 1–3 patients, no correlation is found between tumor size and progression of hearing loss either ipsi- or contralaterally, which supports prior findings that tumor size alone is a poor predictor of hearing loss progression (9, 10).

The age of patients with an additional non-VS schwannoma or meningioma, but not meeting strict NF2 criteria, is significantly higher compared to the unilateral VS group. It is possible that these older patients have simply had more time and opportunity to develop multiple sporadic schwannomas or meningiomas, even without predisposing NF2 mutation. That no difference is seen in either ipsi- or contralateral hearing loss progression in this patient population, compared to patients with unilateral VS only, is reassuring that they are not hiding an occult NF2 mutation, and supports use of current guidelines for limiting NF2 diagnosis to only patients with either bilateral VS, ≥3 schwannomas or meningiomas, or unilateral VS plus known family history.

Given that splitting the VS patients by age, sex or tumor size showed no significant difference in SNHL progression, none of these subgroups seem to be confounders for contralateral hearing loss progression. Directionality and significance of results are shown to be consistent regardless of whether three- or four-tone PTA is used as the primary metric for threshold hearing; this finding is particularly valuable given that three-tone PTA measurements are most closely associated with speech reception thresholds, and thus of highest clinical utility, while mechanisms for progressive SNHL can often first affect only higher-threshold frequencies (36, 37). The high degree of alignment in outcomes, regardless of PTA metric used, supports the clinical value of using either three- or four-tone PTA metrics to assess contralateral hearing in VS patients.

### Limitations

Firstly, due to the retrospective study design, availability of records was not complete for all patients, particularly in ability to assess tumor size in some patients. Audiometric follow-up is also highly variable, limiting granularity of longitudinal tracking of hearing outcomes. As Mass Eye and Ear is a tertiary referral center, patients in many cases may have undergone prior audiometry at outside facilities before initial presentation, and these records were not reliably accessible. Furthermore, for many patients with known VS undergoing long-term clinical surveillance, the contralateral ear was not tested at the same frequency or level of detail as for the ipsilateral ear, leading to the potential for premature censoring of some contralateral ears.

Secondly, during the study period covering 24 years, improvements in MRI resolution could have influenced accuracy of tumor size assessment. However, this is unlikely because the smallest tumor noted is 1.5 mm, still detectable even with a 1.5 T MRI (38), and tumor volumes obtained at 1.5T and 3T almost perfectly correlate for a different intracranial tumor (39).

Thirdly, the diagnosis of VS can only be made with 100% certainty through confirmative pathology, which is not available when watchful waiting or radiotherapy is applied. However, even in these cases the diagnosis of VS is very close to definitive because VSs are the most common tumors in the CPA and they lack distinctive radiographic features of other tumors such as the dural tail of a meningioma (40–43).

### Case Control Group

Of particular interest to this analysis is the selection of appropriate case controls—patients with SNHL but no other known sensorineural otologic history and thus presumed presbycusis, as well as a similar interval for follow-up audiometry compared to the study population. A potential selection bias could be present within the case control group if patients with occult otologic history were included, and if this history were not evident based on superficial review of diagnostic codes alone. To minimize this bias we performed detailed chart review on any case control patients who demonstrated significant change in hearing over time, and replaced those with other otologic histories with “clean” controls for whom the baseline otologic disease burden was equivalent to those of VS-contralateral ears, as detailed in the Methods section. This finding reinforces the continued importance of detailed chart review in the context of large database analyses, since over-reliance on the “face value” of historical diagnosis codes can easily overlook key components to the patient history.

## Conclusions

This study represents to date the largest study tracking long term bilateral hearing outcomes in patients with VS. Our findings definitively demonstrate an increased risk of hearing loss in the VS-contralateral ear in patients with baseline VS-ipsilateral abnormal hearing. No variations in risk of contralateral hearing loss were found based on patient age, sex, tumor size, or degree of VS-ipsilateral hearing loss. In patients with baseline normal VS-ipsilateral hearing, no risk to long-term contralateral hearing was found by any metric or in any subgroup. Patients with baseline abnormal hearing in the VS-ipsilateral ear should be counseled of the increased risk to contralateral hearing, and additional care should be taken to avoid other otologic insults such as audiotrauma or potentially ototoxic medications. Alternately, patients with baseline normal VS-ipsilateral hearing can be reassured that the presence of a unilateral tumor does not predispose to worsening hearing loss in the contralateral ear, however should hearing loss in the VS-ipsilateral ear progress then the risk to hearing in the VS-contralateral ear may increase accordingly.

In light of previous research indicating role of secreted factors that may contribute to accelerated hearing loss progression in VS patients, these results indicate that the composition of these secreted factors do appear to be conducive to long-distance percolation through CSF or blood, although likely with some degradation or dilution, given that the effect on contralateral hearing seen in this study is much slower and less severe than the effects seen in the VS-ipsilateral ear. Immune or inflammatory-mediated processes uniquely affecting the inner ear may also play a role. Understanding key characteristics of these potentially relevant mechanisms, and the interplay between them, can help guide future research to better characterize and evaluate the composition and role played by each of these mechanisms.

## Data Availability Statement

The data analyzed in this study is subject to the following licenses/restrictions: Datasets can be made available upon request to interested parties. The primary dataset includes service dates for certain procedures which are considered protected under HIPAA guidelines, and as such cannot be shared publicly. Requests to access these datasets should be directed to konstantina_stankovic@meei.harvard.edu.

## Ethics Statement

The studies involving human participants were reviewed and approved by Partners Human Research Committee Massachusetts Eye and Ear Infirmary. Written informed consent for participation was not required for this study in accordance with the national legislation and the institutional requirements.

## Author Contributions

KS conceived the project and supervised all the work. SE, ME, and KS designed the study. SE and CR collected data from patient charts. SE wrote code for data analysis. Primary analysis and interpretation were then performed by SE and CR, with input from all authors. SE, CR, and KS wrote the manuscript. All authors edited the manuscript and approved the final version.

## Conflict of Interest

The authors declare that the research was conducted in the absence of any commercial or financial relationships that could be construed as a potential conflict of interest.
